# Incidental cardiac mass: when the past influences the future

**DOI:** 10.1093/ehjimp/qyag114

**Published:** 2026-06-26

**Authors:** Cristina Conte, Ilaria Armellini, Michela Puppato, Alessandro Vit, Leonardo Spedicato, Alessandro Telesca, Luca Rebellato, Igor Vendramin, Daisy Pavoni, Massimo Imazio

**Affiliations:** Cardiothoracic Department, University Hospital Santa Maria Della Misericordia, ASUFC, Piazzale Santa Maria della Misericordia, 15, Udine 33100, Italy; Cardiothoracic Department, University Hospital Santa Maria Della Misericordia, ASUFC, Piazzale Santa Maria della Misericordia, 15, Udine 33100, Italy; Department of Radiology, University Hospital Santa Maria Della Misericordia, ASUFC, Udine, Italy; Department of Radiology, University Hospital Santa Maria Della Misericordia, ASUFC, Udine, Italy; Cardiothoracic Department, University Hospital Santa Maria Della Misericordia, ASUFC, Piazzale Santa Maria della Misericordia, 15, Udine 33100, Italy; Cardiothoracic Department, University Hospital Santa Maria Della Misericordia, ASUFC, Piazzale Santa Maria della Misericordia, 15, Udine 33100, Italy; Cardiothoracic Department, University Hospital Santa Maria Della Misericordia, ASUFC, Piazzale Santa Maria della Misericordia, 15, Udine 33100, Italy; Cardiothoracic Department, University Hospital Santa Maria Della Misericordia, ASUFC, Piazzale Santa Maria della Misericordia, 15, Udine 33100, Italy; Cardiothoracic Department, University Hospital Santa Maria Della Misericordia, ASUFC, Piazzale Santa Maria della Misericordia, 15, Udine 33100, Italy; Cardiothoracic Department, University Hospital Santa Maria Della Misericordia, ASUFC, Piazzale Santa Maria della Misericordia, 15, Udine 33100, Italy

A 53-year-old woman with a history of surgical atrial septal defect closure underwent dual-chamber pacemaker implantation for sick sinus syndrome in 2021 and experienced an acute pericarditis the following year. At routine control, transthoracic echocardiogram incidentally revealed a mediastinal mass (*Figure 1A*). Low-velocity colour Doppler demonstrated systolic–diastolic flow within the lesion, with suspected circumferential thrombosis (*Figure 1B*). Agitated saline contrast echocardiography excluded communication with the right cardiac chambers (*Figure 1C*). Contrast-enhanced ultrasound showed progressive opacification of the cardiac chambers followed by delayed enhancement of the mass (*Figure 1D*). Contrast-enhanced chest computed tomography (CT) excluded other aetiologies and revealed a severe sternal malalignment close to the mass, along with adjacent pericardial thickening (*Figure 1E*). Coronary CT angiography demonstrated kinking of the mid-to-distal right coronary artery (RCA) caused by the mass and the close contact between the sternal wires and the blood supply (*Figure 1F* and *G*). Invasive coronary angiography confirmed active perfusion of the mass from the RCA (*Figure 1H*). The presumed final diagnosis was a coronary pseudoaneurysm with circumferential peripheral thrombosis supplied by the RCA.

**Figure 1:**
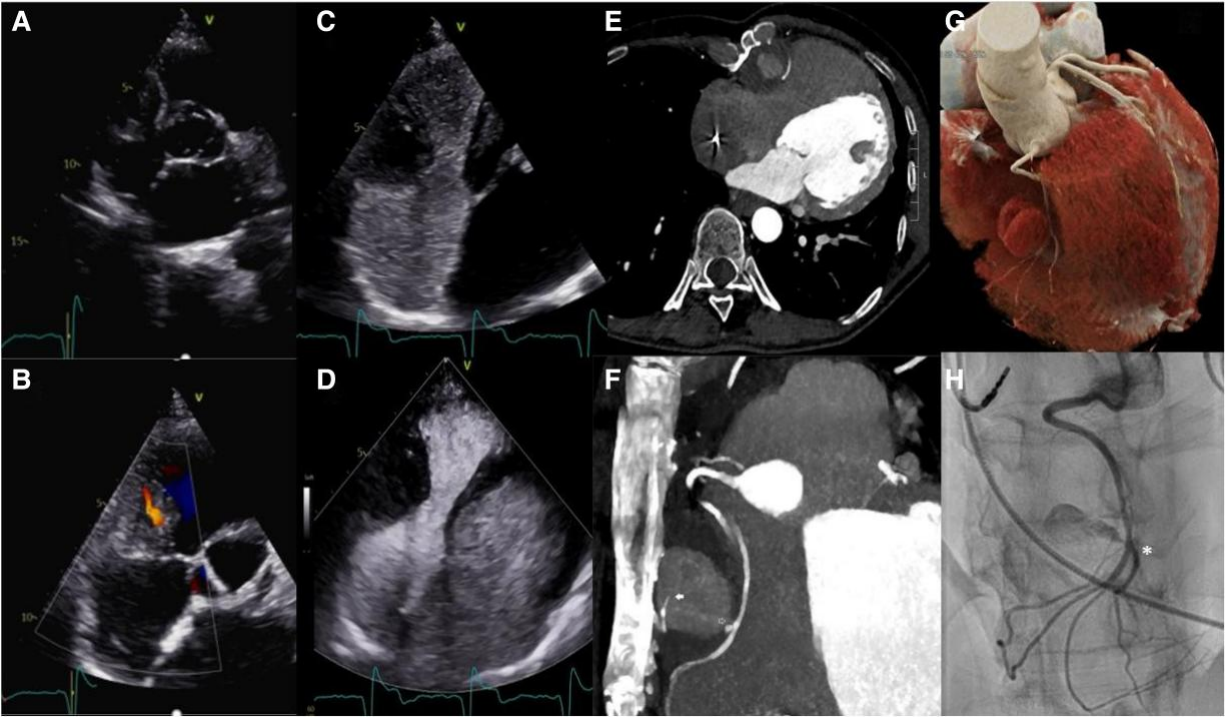
Multimodality approach to mediastinal mass: functional and anatomical consideration. (*A*) Parasternal short-axis view displaying a mediastinal mass adjacent to the right atrioventricular groove. (*B*) Low-velocity colour Doppler application demonstrating systolic–diastolic flow within the lesion, with suspected circumferential thrombosis. (*C*) Agitated saline contrast echocardiography filling the right heart chambers and excluding communication between the right-side heart and the mass. (*D*) Contrast-enhanced ultrasound demonstrating progressive opacification of the cardiac chambers (before right side, then left side), followed by delayed enhancement of the mass, suggesting an arterial supply from the systemic circulation. (*E*) Transversal-axis CT excluding other mediastinal aetiologies, such as pericardial or bronchogenic cysts, displaying the markedly malaligned sternum, the concomitant pericardial thickening, and the secondary close contact between the mass and sternal wire. (*F*) Multiplanar reconstruction (MPR) vertical long-axis view excluding anomalous course of the RCA course (i.e. interatrial course) and confirming the anatomic relationships between the RCA, severely kinked by the mass in the mid-to-distal part, the blood supply, and the sternal wires (white arrows). (*G*) Three-dimensional (3D) volume rendering image clearly displaying the anatomical site of the suspected pseudoaneurysm and the close relationship with the RCA. (*H*) Invasive coronary angiography showing the active arterial blood supply of the mass by the RCA (white *).

Percutaneous options such as covered stent implantation or coil embolization were considered at high-risk of peri-procedural myocardial infarction and infections. Redo surgery was reputed complex given the chest anatomy. Considering the absence of symptoms and haemodynamic impact, a conservative management was chosen by the patient.

We finally hypothesize that the prior episode of suspected pericardial chest pain may have represented the initial manifestation of the erosive vascular process caused by the sternal wires.

## Data Availability

No new data were generated or analysed in support of this research.

## Lead author biography

Cristina Conte, MD, obtained her medical degree and followed cardiology residency at the Catholic University of the Sacred Heart of Rome, Italy. Dr Conte gained extensive experience in the field of cardiac imaging with particular interest in echocardiography, valvular heart disease, and interventional echocardiography. Nowadays, she works in Santa Maria della Misericordia Hospital in Udine, Italy.

